# Urinary Metabolomics Study of the Intervention Effect of Hypoglycemic Decoction on Type 2 Diabetes Mellitus Rats Model

**DOI:** 10.1155/2019/1394641

**Published:** 2019-10-30

**Authors:** Lin-Lin Pan, Qi-Hui Sun, Gui-Rong Liu, Jia-Yin Guo

**Affiliations:** ^1^Department of Chinese Medicine Literature and Culture, Shandong University of Traditional Chinese Medicine, Jinan 250355, China; ^2^Department of Pharmacy, Shandong University of Traditional Chinese Medicine, Jinan 250355, China; ^3^Department of Traditional Chinese Medicine, Shandong University of Traditional Chinese Medicine, Jinan 250355, China; ^4^Guangdong Provincial Key Laboratory of New Drug Screening, School of Pharmaceutical Sciences, Southern Medical University, Guangzhou 510515, China

## Abstract

The hypoglycemic decoction (HD) is a traditional Chinese medicine (TCM) preparation for the treatment of diabetes mellitus (DM), with a remarkable therapeutic effect. However, its mechanism of action is still unclear at the metabolic level. In this study, the biochemical markers from type 2 DM (T2DM) rats, induced by a high-sugar and high-fat diet combined with streptozotocin (STZ), were detected. The metabolomics-based analysis using high-performance liquid chromatography coupled with tandem mass spectrometry (HPLC-MS/MS) was conducted to evaluate urine samples from control, model, metformin, and HD groups. After oral administration of HD for 28 days, the general state, weight, fasting blood glucose (FBG), blood lipid level, oral glucose tolerance test (OGTT), fasting insulin (FINS), insulin sensitivity index (ISI), and homeostasis model assessment of insulin resistance (HOMA-IR) were significantly improved (*P* < 0.01). The western blotting showed that HD can enhance the protein expression of glucose transporter 4 (GLUT4) and adenosine monophosphate-activated protein kinase (AMPK). The metabolomics results revealed that after treatment with HD, the levels of L-carnitine, 1-methyladenosine, 1-methylhistamine, and 3-indoleacrylic acid were upregulated and the levels of riboflavin, phenylalanine, atrolactic acid, 2-oxoglutarate, citrate, isocitrate, cortisol, and glucose were downregulated. The main mechanism may be closely related to the regulation of the tricarboxylic acid (TCA) cycle, phenylalanine metabolism, glyoxylate metabolism, and dicarboxylate metabolism. Additionally, it was also found that HD can regulate the protein expression of GLUT4 and AMPK to interfere with TCA cycle and carbohydrate metabolism to treat T2DM.

## 1. Introduction

Diabetes mellitus (DM) is a complex and serious metabolic disease associated with abnormally high levels of blood glucose attributed to defective insulin secretion or action [1]. Generally, type 2 DM (T2DM) accounts for more than 90% of DM patients, and their body becomes resistant to the normal effects of insulin and/or gradually loses the capacity to produce enough insulin in the pancreas [2]. Recently, the global incidence of DM has increased each year as a consequence of lifestyle changes, population aging, and urbanization, which lead to a series of cardiovascular diseases. It is estimated that by 2035, the number of patients with DM will reach to 600 million, and the incidence of DM in China will increase to 143 million and rank first in the world [3]. According to the International Diabetes Federation, the current yearly cost of DM in China is $25 billion and this cost may continue to increase to over $47 billion in 2030 [4, 5]. Therefore, further research on the pathogenesis of DM and development of reliable and effective therapeutic methods is urgently needed.

Traditional Chinese medicine (TCM) has been widely used to prevent and treat DM for thousands of years and plays an important role in the treatment of T2DM and its complications. According to TCM, the occurrence of DM is related to deficiency of spleen and stomach qi and fire excess from yin deficiency. The TCM hypoglycemic decoction (HD) contains *Astragalus propinquus*, Pueraria, *Rhizoma polygonati*, *Atractylodis Rhizoma*, *Salvia miltiorrhiza*, *Radix Scrophulariae*, *Rhizoma Dioscoreae*, mulberry leaves, *Polygonatum odoratum*, and *Bombyx batryticatus*. It functions by replenishing qi to invigorate the spleen and stomach and nourishing yin to reduce pathogenic fire. Chinese herbal medicines are rich in polysaccharides, terpenoids, peptides, flavonoids, alkaloids, trace elements, and other active ingredients that are beneficial to glycometabolism. Recent pharmacological studies have shown that many active ingredients in this prescription have hypoglycemic effect, as shown in Table 1. To further verify and explore the mechanism of HD in regulating glycometabolism, the specific experiment scheme of this study was designed as follows.

## 2. Materials and Methods

### 2.1. Experimental Animals and Feed Preparation

Healthy male Wistar rats (weight: 200 ± 20 g) obtained from Jinan Pengyue Experimental Animal Breeding Co. Ltd. (SCXK Lu 20180012; Jinan, China). HD, composed of *Astragalus propinquus* (200 g), Pueraria (300 g), *Rhizoma polygonati* (200 g), *Atractylodis Rhizoma* (100 g), *Salvia miltiorrhiza* (200 g), *Radix Scrophulariae* (100 g), *Rhizoma Dioscoreae* (200 g), mulberry leaves (200 g), *Polygonatum odoratum* (200 g), and *Bombyx batryticatus* (200 g), was prepared as a liquid prescription (including raw medicine 3 g/ml, all purchased from Jinan Jianlian Chinese Medicine Co. Ltd., Chinese Herbal Pieces Factory; Jinan, China), which was processed by the Shandong Institute of TCM and then was stored in a sterile container at 4°C.

### 2.2. Reagents and Equipment

The high-performance liquid chromatography coupled with a tandem mass spectrometry (HPLC-MS/MS) system (AB SCIEX, Framingham, MA, USA) used in this study consisted of a ExionLC AD HPLC system (AB SCIEX LLC) and Triple TOF 5600 + Mass Spectrometer detector (AB SCIEX LLC). Other equipment used included a 3–18 K cryogenic high-speed centrifuge (Sigma Laborzentrifugen GmbH, Osterode am Harz, Germany), automatic biochemical analyzer (Vital Scientific, Spankeren, Netherlands), Mettler AE240 electronic balance (Mettler Toledo, Columbus, Ohio, USA), Vortex-Genie2 vortex shaker (Scientific Industries Inc., Bohemia, NY, USA), and electrophoresis apparatus (NO: DYY-6C, DYCZ-24DN; Beijing Liuyi Instrument Factory, China). Reagents used included acetonitrile (NO: 10900830; Merck & Co., Kenilworth, NJ, USA), methanol (NO: 10899607729; Merck & Co.), formic acid (NO: H6070170; CNW technologies GmbH, Duesseldorf, Germany), formic amine (NO: 0000195636; CNW technologies GmbH), total cholesterol (TC), triglyceride (TG), low-density lipoprotein cholesterol (LDL-C), and high-density lipoprotein cholesterol (HDL-C) kit (Nanjing Jiancheng Institute of Bioengineering, Nanjing, China). Fasting insulin (FINS) kit (NO: 20161116; Wuhan Huamei Bioengineering Co., Ltd., Wuhan, China), streptozotocin (STZ) (NO: S0130-1g; Sigma-Aldrich, Saint Louis, MO, USA), citric acid-sodium citrate buffer (No.: PH1716; Fuzhou Feijing Biotechnology Co., Ltd., Fuzhou, China), metformin hydrochloride tablets (No.: AAH5779; Sino-US Shanghai Squibb Pharmaceutical Co., Ltd., Shanghai, China), rat feed (Beijing Keao Xieli Feed Co., Ltd., Beijing, China), blood glucose meter, and blood glucose test strip (NO: 20162402313; Roche Diagnostics GmbH, Rotkreuz, Switzerland) were also used.

### 2.3. Establishment of the T2DM Rat Model

The T2DM model was induced by the method of Zhang et al. [29, 30]. After adaptive feeding for 1 week, the rats were randomly divided into two groups, namely, the T2DM model rats and control rats. The T2DM model rats were established with a high-sugar and high-fat diet consisting of 67% rat maintenance feed + 10% lard + 20% sucrose + 2.5% cholesterol + 0.5% sodium cholate (mainly consisting of 25% fat, 45% carbohydrate, and 20% protein). The control rats were given a regular diet. After 8 weeks of dietary intervention, the T2DM model rats were injected intraperitoneally with 50 mg/kg STZ (0.1 M, pH 4.5) twice, 2 days in a row. In contrast, the control rats were injected with citrate buffer (vehicle, pH 4.5) with a dose volume of 1 ml/kg. Subsequently, blood samples were collected by tail cutting for fasting blood glucose (FBG) measurements using blood glucose test strips. The blood glucose levels >16.7 mmol/L after STZ-injection was used as the T2DM model rats, and the failed rats were excluded from the study.

### 2.4. Grouping and Administration

T2DM rats were randomly divided into three groups: the model group (fed with saline according to the body weight, 0.5 ml/100 g), metformin group (fed with metformin 0.5 ml/100 g, at a concentration of 0.1 g/kg), and HD group (fed with a HD solution, at 0.5 ml/100 g, the doses was based on clinical experience and a transforming formula = (daily dose of HD in humans (g))/human weight (kg))  × 9.1, the usual human weight is 60 kg in Asia and 40 g/d is the daily dose of HD). Each group consisted of 8 rats and received intragastric administration for 28 days. During the administration period, the rats in the control group were fed with maintenance diet (mainly consisting of 5% fat, 55% carbohydrate, and 25% protein), while the other groups were fed with high-sugar and high-fat diet.

### 2.5. Sample Preparation

#### 2.5.1. Urine Samples for Metabonomics

After the treatment for 28 days, each rat was placed individually in a metabolic cage, and after 24 hours, urine samples were collected and stored in a refrigerator at −80°C for refrigeration. Before testing, the urine samples were placed at −20°C for 30 min and then in a refrigerator at 4°C to thaw until no ice was visible in the sample. Next, 100 *μ*L of the urine sample was placed in an Eppendorf tube, followed by the addition of 300 *μ*l of methanol (containing the internal standards chloramphenicol and clenbuterol), and then vortexed for 1 min and centrifuged at 14,000 g for 30 min. Eventually, 10 *μ*l of the supernatant was placed into a new Eppendorf tube for analysis, and each sample was mixed into a quality control (QC) sample for HPLC-MS/MS analysis.

#### 2.5.2. Serum Samples for Biochemical Index

After the treatment for 28 days, all rats were fasted for 8 hours, the blood samples were collected by tail cutting for FBG measurements using blood glucose test strips, and the blood glucose of tail tips was measured as the blood glucose level at 0 h. Then, a 2 g/kg glucose solution was orally administered to each rat, and the blood glucose levels were measured at 0.5, 1, and 2 hours after glucose administration. The rats were fasted for 12 h before the last administration, blood was collected from the abdominal aorta and centrifuged in a cryogenic centrifuge at 3,500 g/min for 15 minutes, and then it was stored at −80°C for biochemistry analysis tests. The contents of TC, TG, HDL-C, and LDL-C in rat serum were determined according to the kit instructions. The FINS and oral glucose tolerance test (OGTT) of rats were determined by enzyme-linked immunosorbent assay (ELISA), and the insulin sensitivity index (ISI) and homeostasis model assessment of insulin resistance (HOMA-IR) were calculated using the appropriate formulas.

#### 2.5.3. Hepatic Tissue for Western Blotting

Hepatic tissues were prepared for protein extraction. Total protein was acquired by using RIPA buffer (NO: G2002, Servicebio, China) with PMSF (G2008, Servicebio) for 30 min followed by centrifugation at 12000 rpm for 10 min at 4°C. Next, the nuclear protein was obtained by using a Nuclear Protein Extraction kit (G2007, Servicebio), the concentrations were determined by using BCA protein assay (G2026, Servicebio), the total protein was subjected to SDS-PAGE gel electrophoresis (G2003, Servicebio) and electroblotted onto PVDF membranes (ISEQ00010, Millipore, USA), and the membranes were blocked for 1 h in 5% BSA (G5002, Servicebio) and subsequently incubated with the following primary antibodies at 4°C overnight. Primary antibodies were ACTIN (mouse monoclonal antibody 1:1000, GB12001, Servicebio), GLUT4 (rabbit monoclonal antibody 1:1000, bs-0384, BIOSS, China), and AMPK (rabbit monoclonal antibody 1:1000, GB11627, Servicebio), and then, the membrane was incubated with the secondary antibody 1:3000 and the conjugates were visualized with an ECL system (G2014, Servicebio). Finally, the odyssey imaging system was used to analyze protein expression.

### 2.6. Chromatography and Mass Spectrometry Conditions

All samples were analyzed using the HPLC-MS/MS system following the manufacturer's instructions. First, all chromatographic separations were performed using an HPLC system. The sample was collected on a Waters BEH (100 *∗* 2.1 mm, 1.7 um column). The column oven was maintained at 40°C, and the flow rate was 0.4 ml/min. In the positive ion mode, the mobile phase consisted of solvent A (water + 0.1% formic acid) and solvent B (organic phase acetonitrile). In the negative ion mode, the mobile phase consisted of solvent A (0.1% formic acid +5 mM ammonium acetate) and solvent B (organic phase acetonitrile). The specific chromatographic conditions are listed in Table 2.

An AB SCIEX Triple TOF 5600 + mass spectrometer was used to collect data in the positive and negative ion modes separately. The scanning method was a classic data-dependent scanning (IDA), and one primary mass spectrometry scan (100 ms) triggers 10 secondary mass spectrometry scans (500 ms). Dynamic background deduction (DBS) was turned on, first-level scanning range: 50 m/z–1000 m/z; secondary scanning range: 50 m/z–1000 m/z; air curtain gas: 35 psi; atomizing gas: 55 psi; auxiliary atomizing gas: 55 psi; ion source temperature: 550°C; decluster voltage: 80 V; and collision voltage: 35 ± 15 V.

### 2.7. Data Analysis

The total ion current (TIC) data collected by HPLC-MS/MS were derived from the MassLynx 4.1 workstation. The raw data were converted to contain *t*-m/z ion pairs. The data matrix of the sample name and peak intensity was processed by peak matching, peak alignment, controlled processing, and multivariate statistical analysis. Partial least-squares discriminant analysis was used to obtain the score plot by SIMCA-P12.0. By combining discriminant analysis and multivariate statistical methods, several of the most significant metabolic markers were selected from the massive data sets. The variable importance projection (VIP) values obtained from the PLS-DA model were used to select the different variables with greater contribution, and then, the *t*-test was performed. The metabolites with VIP > 1 and *P* < 0.05 were used as potential biomarkers. The differential mass-to-charge ratios were identified by searching the Human Metabolome Database (HMDB) (http://www.hmdb.ca), METLIN metabolomics database (http://www.metlin.scipps.edu), and the laboratory self-built database, and then the relevant metabolic pathways were analyzed.

## 3. Results

### 3.1. General State and Weight Observation

**Figure 1:**
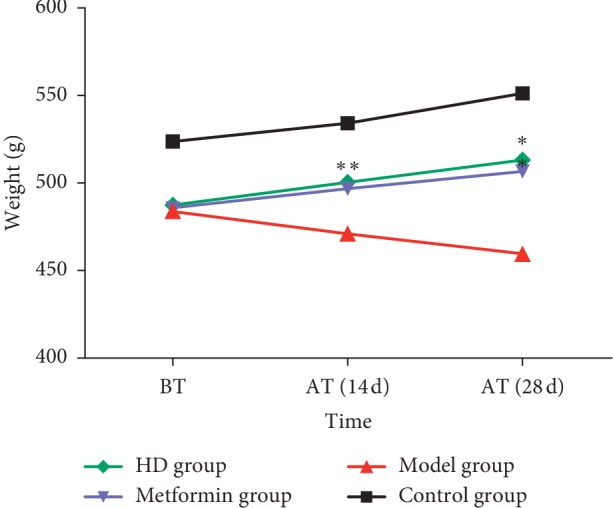
Changes in body weight of rats in each group. Note: data are means ± SD, compared with the model group in the same period of time, ^*∗*^*P* < 0.01; BT: before treatment; AT: after treatment.

The rats in the control group had a normal feeding, drinking, excretion, and weight gain, as well as smooth and shiny hair. In the model group, the rats had a gradually decreased weight (*P* < 0.01), obviously increased drinking, feeding, and excretion, moist cage, and dirty and messy fur. After treatment, the general states of the rats in the metformin group and HD group were improved, and as shown in Figure 1, their body weight gradually increased compared with that before treatment (*P* < 0.01).

### 3.2. Blood Lipid Level

**Figure 2:**
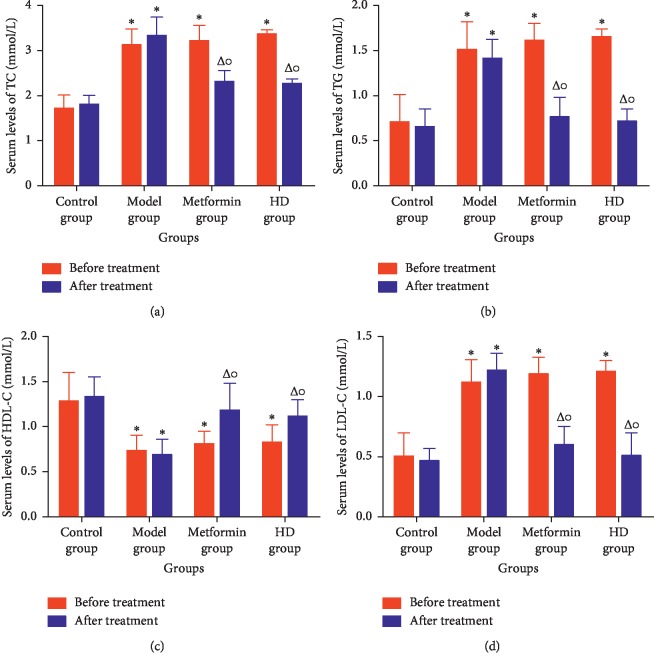
Blood lipid level of each group. Note: compared with the control group at the same time period, ^*∗*^*P* < 0.01; compared with the model group at the same time period, ^Δ^*P* < 0.01; compared with its own group before treatment, ^○^*P* < 0.01. (a) TC. (b) TG. (c) HDL-C. (d) LDL-C.

As shown in Figure 2, before treatment, the serum levels of TC, TG, and LDL-C in the model group were significantly higher than those in the control group (*P* < 0.01), and the HDL-C level in the model group significantly decreased (*P* < 0.01). After treatment, the serum levels of TC, TG, and LDL-C in the metformin and HD groups were significantly lower than those in the model group (*P* < 0.01), and the HDL-C level also significantly increased (*P* < 0.01), indicating that HD acts by regulating the blood lipid levels.

### 3.3. Analysis of FBG, OGTT, FINS, ISI, and HOMA-IR

**Figure 3:**
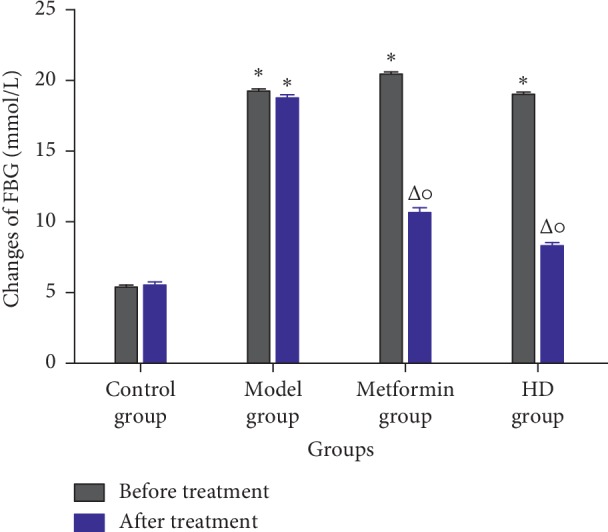
Changes of FBG in each group (*x* ± *s*). Note: compared with the control group at the same time period, ^*∗*^*P* < 0.01; compared with the model group at the same time period,△ *P* < 0.01; compared with its own group before and after treatment, ^○^*P* < 0.01.

As shown in Figure 3, compared with the control group before treatment, the FBG of rats from the model, HD, and metformin groups increased significantly (*P* < 0.01), indicating that the T2DM model of rats was successfully developed. Compared with the model group after treatment, the FBG of the metformin and HD groups decreased significantly (*P* < 0.01). At the same time, the comparisons between the same group before and after treatment revealed that the FBG in the metformin and HD groups significantly decreased after treatment (*P* < 0.01), indicating that HD has a good hypoglycemic effect on T2DM rats.

**Figure 4:**
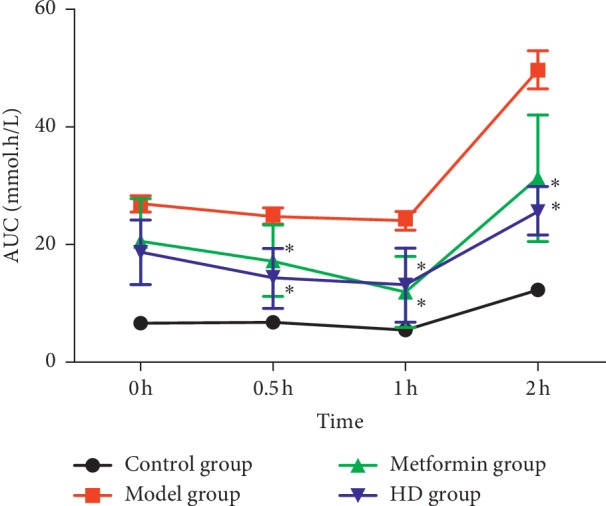
OGTT levels of each group at different time periods. Note: compared with the model group, ^*∗*^*P* < 0.01.

**Figure 5:**
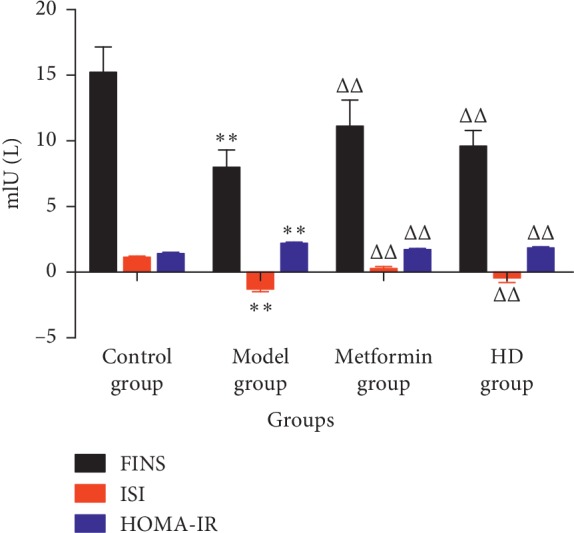
FINS, ISI, and HOMA-IR levels of each group. Note: compared with the control group, ^*∗∗*^*P* < 0.01; compared with the model group, ^ΔΔ^*P* < 0.01.

As shown in Figure 4, at 0.5, 1, and 2 h, the OGTT levels in the metformin and HD groups were significantly lower than those in the model group (*P* < 0.01). Also, at 0.5 and 2 h, the level of OGTT in the HD group was lower than that in the metformin group, indicating that HD had a good effect in reducing the level of OGTT in T2DM rats. As shown in Figure 5, the level of FINS in the metformin and HD groups was significantly higher than that in the model group (*P* < 0.01). Additionally, the values of the ISI and HOMA-IR indicators, which were calculated by the formulas: ISI = 1n [1/(FBG × FINS)] and HOMA-IR = 1n  [(FINS × FBG)/22.5], respectively, also increased significantly compared with those in the model group (*P* < 0.01).

### 3.4. Western Blotting

**Figure 6:**
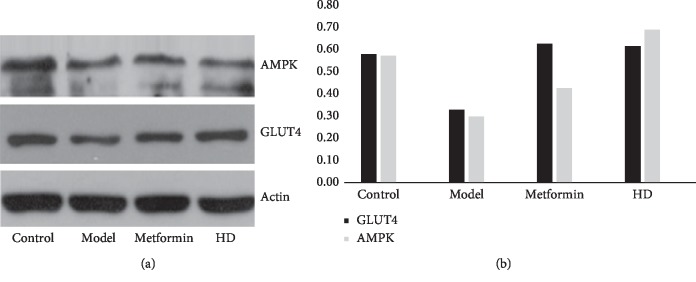
(a) Protein expression of GLUT4 and AMPK in each group. (b) refers to the ratio of the gray value of the target strip to the internal reference strip.

The level of glucose is a key indicator for assessing DM, and the protein glucose transporter 4 (GLUT4) and adenosine monophosphate-activated protein kinase (AMPK) play important roles in glucose [31, 32], so we analyzed the protein expression of GLUT4 and AMPK in each groups. As shown in Figure 6, the protein expressions of GLUT4 and AMPK were significantly decreased in the model group, while the HD and metformin treatment obviously facilitated the GLUT4 and AMPK expression compared with the model group.

### 3.5. Metabolomic Analysis

**Figure 7:**
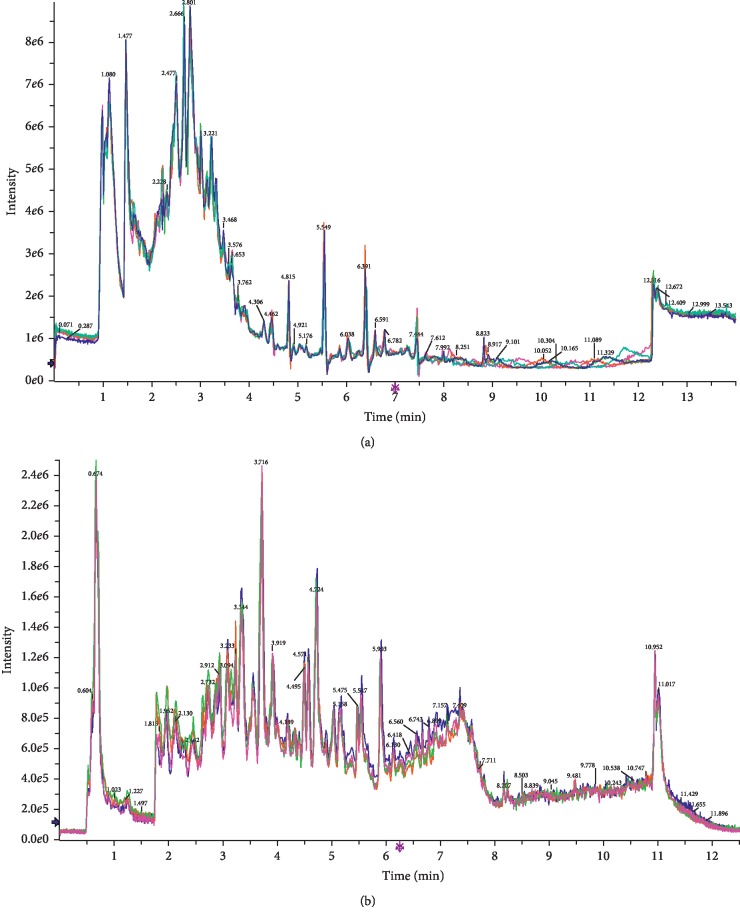
Total ion flow chart. (a) Total ion flow chart of sample in the positive ion mode; (b) Total ion flow chart of sample in the negative ion mode.

**Figure 8:**
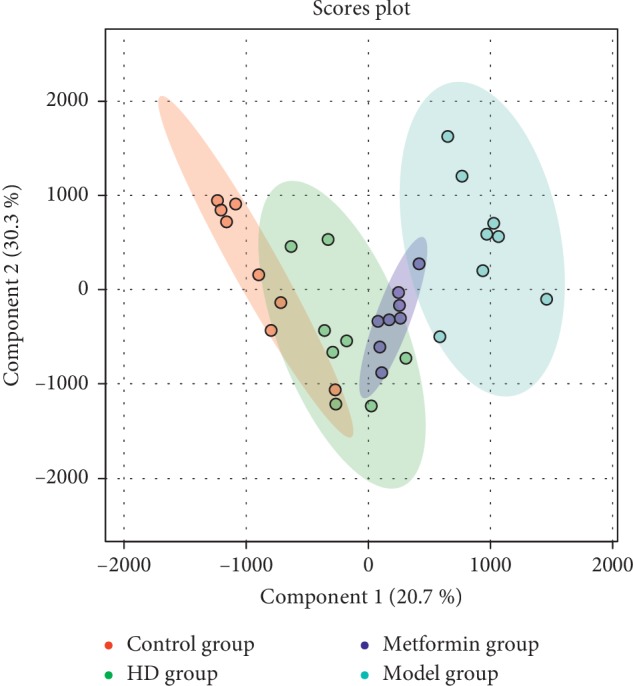
OPLS-DA score plot of each group.

As shown in Figure 7, the overlaid chromatograms of the QC show good reproducibility for the retention time and peak (shape), indicating that the chromatographic separation and apparent reproducibility were well. The relative standard deviation (RSD) of the peak area of the internal standards chloramphenicol and clenbuterol was less than 5%, indicating that the repeatability of the detection method meets the requirement. Principal component analysis (PCA) is an unsupervised model analysis method that is mainly used to assess the trend of separation and variation between groups and within groups or whether abnormal points exist. The orthogonal partial least-squares discriminant analysis (OPLS-DA) method is a supervised discriminant analysis statistical method that can best reflect the differences between classification groups. As shown in Figure 8, the OPLS-DA score plot shows that the intergroups were well separated, indicating that the sample modelling was satisfactory. Moreover, according to the discrete degree of each group, the HD group was closer to the normal group after treatment, followed by the metformin group, and lastly, the model group, indicating that the HD has a better therapeutic effect than the other drugs evaluated.

**Figure 9:**
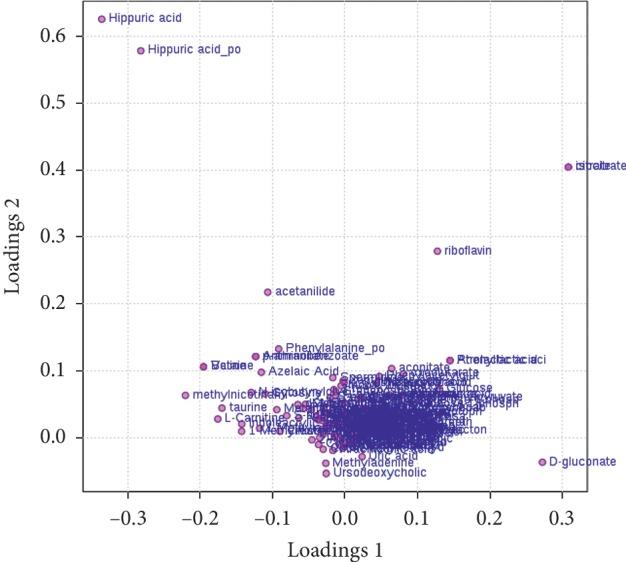
Loading plot. Note: The farther away a substance is from the centre (origin) in the figures, the greater is the difference in the substance between the different groups.

**Figure 10:**
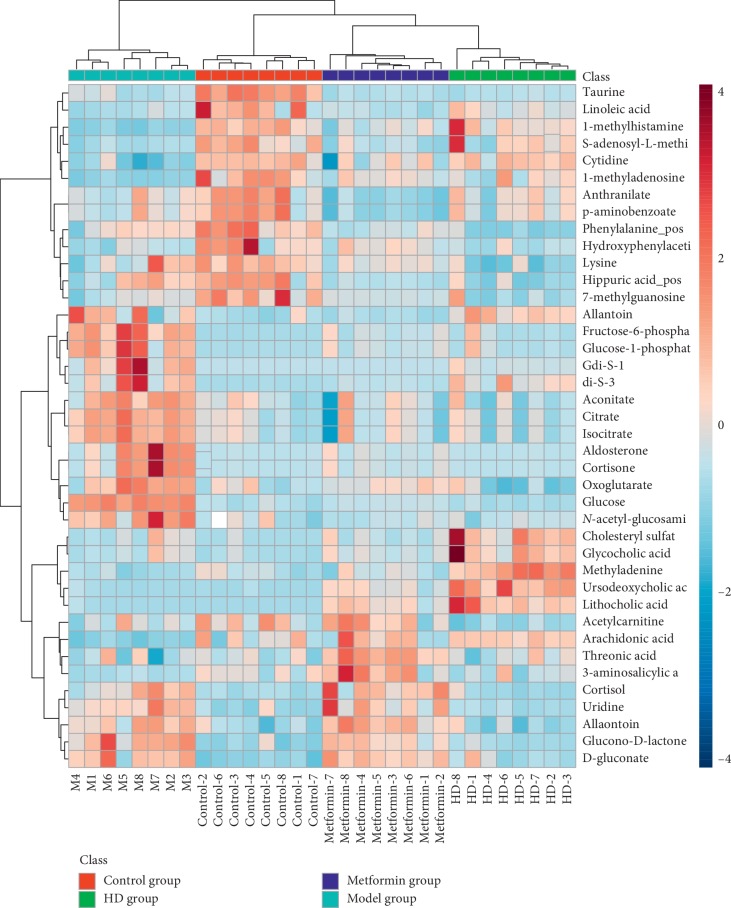
Cluster heat plot.

**Figure 11:**
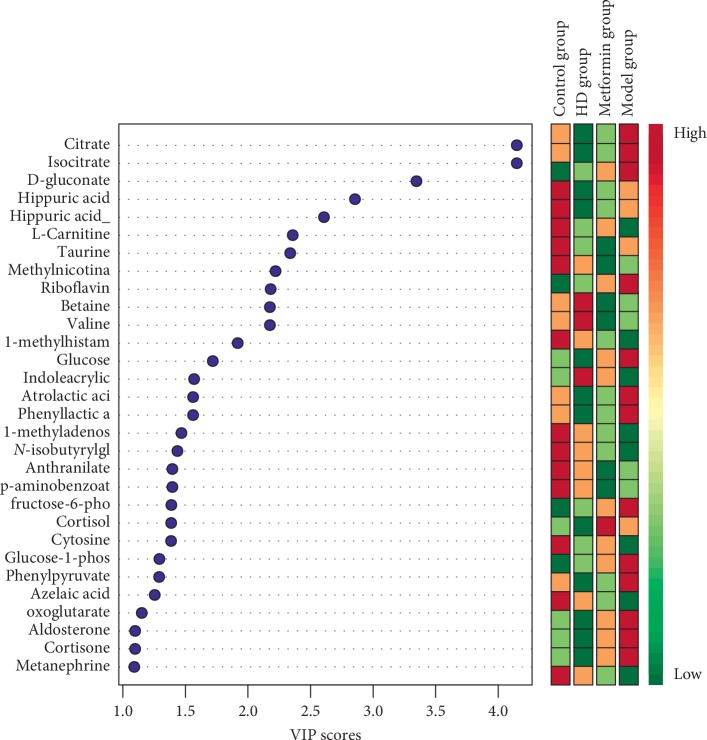
VIP plot.

The mass-to-charge ratio with large dispersion in the statistical analysis of the loading plot plays an important role in the separation between groups, as shown in Figure 9, the loading plot identified the metabolites with significant differences in abundance between groups. As shown in Figure 10, the cluster heat plot clearly reveals the different components among different groups. The VIP value represents the contribution of each measured variable to the separation of samples. Generally, when the VIP value was larger, the contribution was greater. As shown in Figure 11, The VIP plot shows that the VIP value of many metabolites was greater than 1; thus, we can screen out these substances for further research.

**Figure 12:**
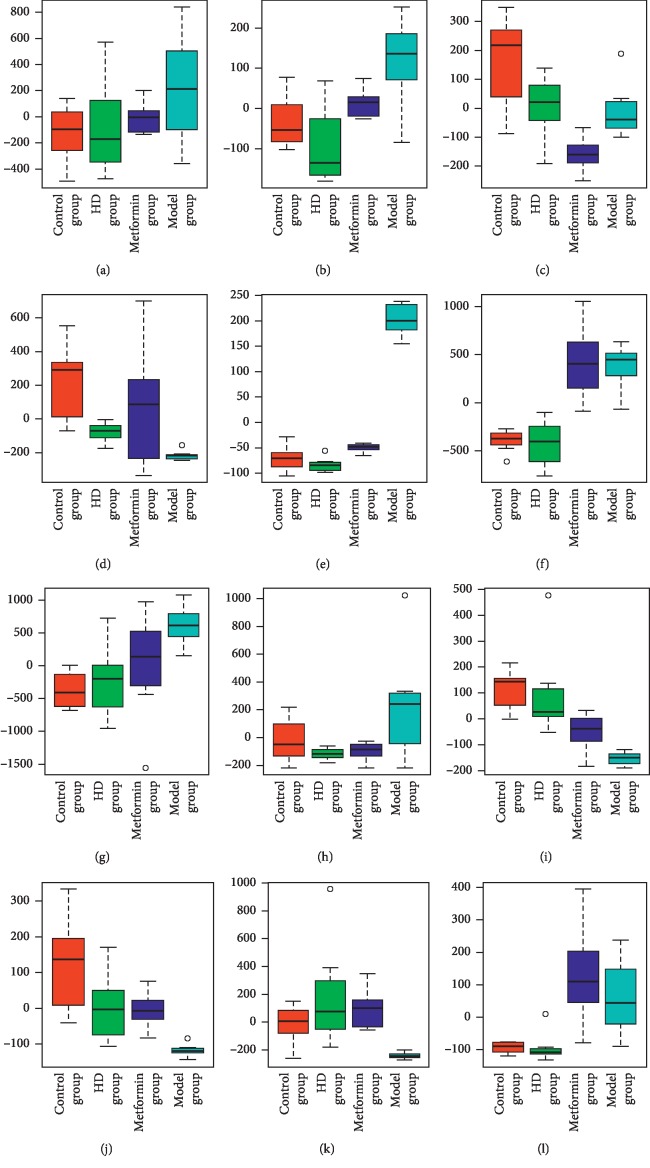
Comparison of the relative intensity of potential metabolites. (a) Riboflavin. (b) 2-Oxoglutarate. (c) Phenylalanine. (d) L-Carnitine. (e) Glucose. (f) Citrate. (g) Isocitrate. (h) Atrolactic acid. (i) 1-Methylhistamine. (j) 1-Methyladenosine. (k) 3-Indoleacrylic acid. (l) Cortisol.

**Figure 13:**
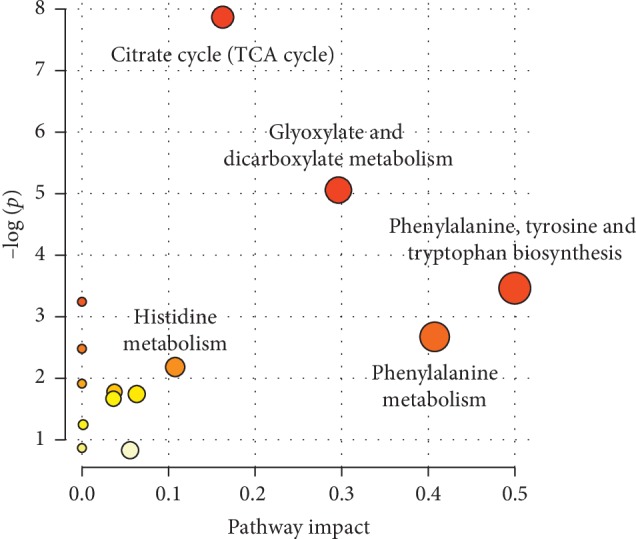
Metabolic pathway analysis of differential metabolites. Note: the dots represent the pathways that were matched using pathway impact values from pathway topology analysis and *p* values from pathway enrichment analysis. Colors (varying from yellow to red) represents the metabolites in our data are with different levels of significance for enrichment analysis.

In this study, the multivariate data analysis was performed on the resulting metabolite profile, in which different metabolites were screened according to the VIP value > 1 and *P* < 0.05. The potential biomarkers were searched in the Kyoto Encyclopedia of Genes and Genomes (KEGG) database (http://www.kegg.com) based on the retention time and exact mass-to-charge ratio of the variables. As shown in Table 3, 12 potential biomarkers were identified, and their specific content changes are shown in Figure 12. It was determined that after treatment with HD, the biomarkers of L-carnitine, phenylalanine, 1-methyladenosine, 1-methylhistamine, and 3-indoleacrylic acid were upregulated in the urine and riboflavin, atrolactic acid, 2-oxoglutarate, citrate, isocitrate, cortisol, and glucose were downregulated. As shown in Figure 13, the impacts on the pathways tricarboxylic acid (TCA) cycle, phenylalanine metabolism, glyoxylate, and dicarboxylate metabolism were stronger.

## 4. Discussion

### 4.1. Biochemical Research

Metformin is a first-line antidiabetic drug and has been used in clinical practice for decades. On the one hand, the previous studies indicated metformin can act on lipid metabolism [33] and reduce the blood lipid level in mice fed with high-fat diet, and the mechanism is related to the protein expression, the p-AMPK/AMPK ratio, and a key kinase-regulating cellular energy homeostasis [34], As shown in Figure 2, the blood lipid level in metformin and HD groups obviously improved, indicating that metformin and HD have a good lipid-lowering effect. On the other hand, metformin can increase the protein expression of the GLUT4 [35] and AMPK [36], which are closely related to glucose. In this study, the protein expression of the GLUT4 and AMPK increased obviously after treatment with HD and metformin; this is not only consistent with previous research results but also indicates that HD has a similar mechanism to metformin in increasing the protein expression of the GLUT4 and AMPK to regulate the glucose in the treatment of T2DM. In addition, the biochemical analysis revealed that after the treatment with HD and metformin, the FBG, OGTT, FINS, ISI, and HOMA-IR, which are closely related to glucose significantly improved, and compared with the metformin group, some biochemical markers, such as HDL-C, FBG, OGTT, and FINS, were better regulated in the HD group.

### 4.2. Metabolomics Research

**Figure 14:**
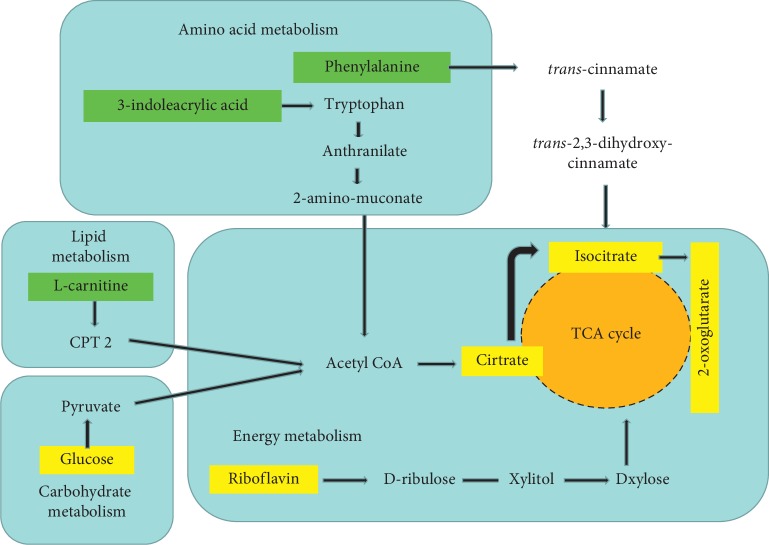
Potential metabolic pathways disturbed in T2DM rats after treatment with HD. Note: the blue frame represents the metabolic pathway, the green frame represents the rise of potential biomarkers, and the yellow frame represents the decline of potential biomarkers.

According to the metabolomics analysis, HD can effectively regulate 12 metabolites. Also, compared with the metformin group, the levels of riboflavin, glucose, 2-oxoglutarate, citrate, cortisol, isocitrate, phenylalanine, 1-methyladenosine, and 1-methylhistamine in the HD group were closer to those in the control group, indicating that HD has a more obvious advantage over metformin in regulating these metabolites associated with T2DM. Moreover, HD can affect the metabolism of amino acids, energy, carbohydrate, and the lipid pathway (Figure 14), and the impacts on the pathways of the TCA cycle, phenylalanine metabolism, and glyoxylate and dicarboxylate metabolism were relatively stronger.

#### 4.2.1. Energy Metabolism

The TCA cycle is a common pathway for the complete oxidation of acetyl coenzyme A (Acetyl-CoA); it can release energy from sugars, fats, and proteins (amino acids) and is also the hub for their interconnection and transformation [37]. Citrate is an important intermediate product in the TCA cycle and is also the terminal metabolic pathway and metabolic linkage of lipid biosynthesis. Previous studies found that citrate was significantly increased in the DM rats and liquid citrate acidification can most effectively inhibit glycolysis [38, 39], which are consistent with our finding that the level of citric acid increased in the model group and decreased in the HD group. Citric acid can reversibly produce isocitrate under the action of aconitase, and the isocitrate dehydrogenase (ICD) is a key rate-limiting enzyme in the TCA cycle. Another previous study found that the activity of ICD in DM rats was lower than that in the control group rats [40], which indicates that DM can inhibit the activity of ICD.

The metabolite 2-oxoglutarate is located at the intersection between the carbon and nitrogen metabolic pathways and is a key molecule in the TCA cycle, playing a crucial role in determining the overall rate of this metabolic process. In the TCA cycle, 2-oxoglutarate is decarboxylated to succinyl-CoA and carbon dioxide by 2-oxoglutarate dehydrogenase, which functions as a key control point of the TCA cycle [41]. Additionally, 2-oxoglutarate can be generated from isocitrate by oxidative decarboxylation catalyzed by ICD, and in this study, the change trend of 2-oxoglutarate was consistent with that of isocitrate. Moreover, 2-oxoglutarate can significantly influence circulating plasma levels of hormones, such as insulin, growth hormone, and insulin-like growth factor-1 [41].

As shown in Figure 14, D-ribulose, xylitol, and D-xylose can be generated from riboflavin and then act on the TCA cycle. Riboflavin, which is also known as vitamin B2, is involved in biological oxidation and energy metabolism and is related to the metabolism of carbohydrates, proteins, nucleic acids, and fats. Oxidative stress plays a major role in the pathogenesis of T2DM, and riboflavin can reduce the risk of T2DM complications by regulating oxidative stress [42]. In this study, as an exogenous substance, the riboflavin level in the urine of HD group was significantly reduced compared with the model group, so HD may increase the absorption or utilization of riboflavin in the body.

#### 4.2.2. Lipid Metabolism

L-Carnitine is an amino acid which plays an important role in lipid metabolism by regulating fatty acid transport between the cytosol and mitochondria [43]; humans can obtain both by food ingestion and endogenous synthesis from trimethyl-lysine [44]. In fact, previous studies have shown that L-carnitine deficiency can impair insulin sensitivity and cause elevation of fasting glucose [45]. Also, supplementation with L-carnitine can reduce oxidized LDL-C levels in patients with T2DM [46]. Additionally, L-carnitine can also improve the quality of life by reducing the frequency and severity of muscle cramps in DM patients [47]. In our study, an increased level of L-carnitine was observed in the HD groups, indicating that HD can normalize the level of L-carnitine in the body.

Cortisol, which is one of the glucocorticoids extracted from the adrenal cortex, is an adrenocortical hormone with the strongest effect on glycometabolism. Previous studies found that the level of cortisol plays an important role in the development of T2DM [48]. The association of cortisol with T2DM was shown when it was found that there was a significant upward trend in the cortisol level before bedtime in T2DM patients [49], and the circadian rhythm of cortisol secretion was found to be the main determinant of glycemic control in humans [50]. This study showed that HD can regulate the level of cortisol to be consistent with the control group after treatment.

#### 4.2.3. Amino Acid Metabolism

Phenylalanine is an essential aromatic amino acid in humans (provided by food) and plays a key role in the biosynthesis of other amino acids. In vivo, most of the phenylalanine is oxidized to tyrosine catalyzed by phenylalanine hydroxylase, and together with tyrosine, they synthesize important neurotransmitters and hormones, which participate in the metabolism of body sugar and fat [51]. As a nutrient supply substance, phenylalanine can promote insulin secretion [52]. In our study, the level of phenylalanine increased obviously, and the pathway impact of phenylalanine metabolism also plays an important role as shown in Figure 13.

#### 4.2.4. Carbohydrate Metabolism

Glucose is a primary source of energy for living organisms. In animals, glucose is synthesized in the liver and kidneys from noncarbohydrate intermediates, such as pyruvate and glycerol, by a process known as gluconeogenesis. DM patients suffer from insulin deficiency, which can lead to reduced glucose decomposition and increased glycogen decomposition and gluconeogenesis [53]. However, excessive glucose can be converted into fatty acids and triglycerides in the liver and adipose tissue and increase insulin concentration, thereby inducing obesity and DM. In this study, the level of glucose was clearly decreased in the HD and metformin groups, indicating that HD has a significant effect in reducing glucose.

#### 4.2.5. Other Findings

Mitochondria is an organelle that generates energy in cells, and 1-methyladenosine is a methylated nucleotide-containing purine base in human mitochondria, which occurs at position 58 of tRNALeu (UUR) [54]. tRNA is a core component of protein synthesis and cellular signaling networks. Demethylation of 1-methyladenosine in tRNA affects protein synthesis and glucose utilization [55]. In this study, an increased level of 1-methyladenosine was observed in the HD and metformin groups, and the trend is closer to the control group than the model group. In addition, the level of 1-methylhistamine, 3-indoleacrylic acid, and atrolactic acid was also increased, but there was no research showing the function of them in the occurrence, development, or treatment of T2DM, so whether they can be used to treat T2DM still needs further verification.

### 4.3. Relevance of the Potential Mechanisms

**Figure 15:**
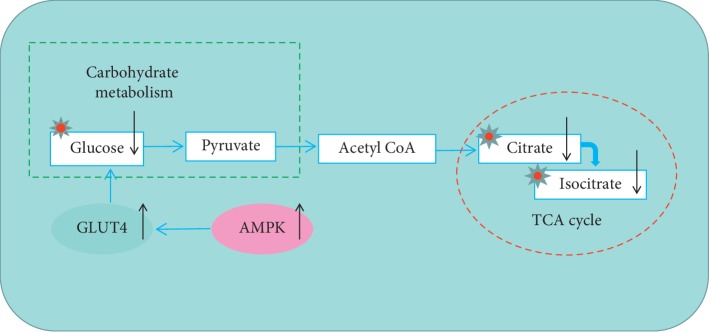
Regulation of metabolism by related proteins in T2DM rats after treatment with HD. Note: the ↑ represents an upward trend; ↓ represents a downward trend.

The metabolite glucose uptake in adipose tissue and muscle is mostly mediated by GLUT4 and has a critical function in glucose utilization by insulin stimulation [35]. Hepatic glucose uptake and glucose production are the key parameters in glucose homeostasis, and AMPK plays an important role in this process with the function of suppressing gluconeogenesis in the liver and promoting glucose uptake in peripheral tissues [36]. Previous studies have found that the activation of AMPK signal pathways can regulate the activity and translocation of GLUT4 and facilitate glucose absorption [56]. In addition, it is known that the contribution of glucose to tissue TCA metabolism is primarily indirect in all tissues [57], so changes in glucose levels can also affect TCA cycles. As shown in our study (Figure 15), the expression of protein GLUT4 and AMPK increased significantly, the metabolite glucose in the urine showed a downward trend, and the metabolic pathway TCA cycle and its metabolites (citrate and isocitrate) also showed obvious reactions. The above evidence proves that HD can regulate the protein expression of GLUT4 and AMPK to interfere with TCA cycle and carbohydrate metabolism in the treatment of T2DM.

## 5. Conclusion

HD embodies the characteristics of balancing Yin and Yang and regulating qi, blood, and body fluid. This study showed that HD has a good regulatory effect on glycometabolism in T2DM rats by improving the biochemical markers related to blood glucose. In metabolomics analysis, the treatment of T2DM with HD led to normalisation of levels in TCA cycle and carbohydrate metabolism by regulating the protein expression of GLUT4 and AMPK. Therefore, HD can be used as a potential drug to treat T2DM from multiple links and targets.

## Figures and Tables

**Table 1:** Active ingredients of HD in the treatment of T2DM.

Medicine	Active ingredient	Effect
*Astragalus propinquus*	Astragalus polysaccharides (APS)	Restoring impaired insulin signaling in insulin-resistant rats and improving insulin resistance [6]
	APSI, APSII, APSIII Dextran	Increasing insulin sensitivity and lowering glucose [7, 8]

Pueraria	Puerarin	Lowering the serum TC level and inhibiting arteriosclerosis [9]
	Flavone	Having significant hypolipidemic and antioxidant effects, and it may be beneficial to the prevention of atherosclerosis [10]

*Rhizoma polygonati*	The decoction	Lowering the TC, TG, *β*-lipoprotein, and blood cholesterol levels [11]
	Polysaccharides	Inhibiting the oxidation of the liver lipid; regulating the expression level of the corresponding genes and proteins relating to the lipid metabolism [12]

*Atractylodis Rhizoma*	Polysaccharides	Having an antidiabetic effect, and the mechanism might be related to its antioxidant activity [13]
	Atractylenoide	Inhibiting the proliferation and capillary formation of human umbilical vein endothelial cells and preventing the occurrence of diabetic retinal complications [14]

*Salvia miltiorrhiza*	The injection	Reducing TLR4 protein expression in NRK-52E cells to improve DM [15]
	Magnesium lithospermate B	Anti-inflammation, antioxidation, and antiatherosclerosis [16]
	Caffeic acid	Antioxidation, lowering blood pressure, and preventing cardiovascular and cerebrovascular diseases [17]
	Tanshinone IIA sodium sulfonate	Enhancing the total antioxidant capacity and exerting protective effects on the myocardium of DM rats [18]
	Salvianolic acids	Regulating the damage of gene expression in brain tissue of DM rats [19], upregulating PPAR-α expression, and improving insulin resistance [20]

*Radix Scrophulariae*	Polysaccharides	Improving the metabolisms of sugar and fat, enhancing the antioxidant activity and increasing insulin, and reducing blood glucose level [21]

*Rhizoma Dioscoreae*	Polysaccharides	Having a protective effect on renal function in DM mice, and its mechanism may be related to the inhibition of high glucose-activated AR/P38MAPK/CREB signaling pathway [22]
	Polyphenols and saponins	Lowering blood glucose level [23]

Mulberry leaves	Flavonoids, alkaloids, polysaccharides extracts	Upregulating 4-hydroxydihydrosphingosine, which regulates lipid metabolism [24]
	1-Deoxynojirimycin	Promoting the proliferation of islet *β*-cells [25] and regulating blood glucose level

*Polygonatum odoratum*	Polysaccharides	Inhibiting the expression of P-JNK and p65nf-kappa B protein, reducing oxidative stress and inflammation, and increasing insulin secretion, thereby lowering blood glucose level [26]
	Total saponins	Reducing blood glucose by inhibiting the activity of *α*-glucosidase [27]

*Bombyx batryticatus*	The decoction	Treating DM by increasing glucose utilization in the body [28]

**Table 2:** Chromatographic conditions in the positive and negative ion mode.

Condition	Time (min)	The organic phase B (%)
Positive	1.0	10
	9.0	95
	12.0	95
	12.1	10
	14	Stop

Negative	1.0	10
	7.5	95
	10.0	95
	10.1	10
	12.5	Stop

**Table 3:** List of different metabolites.

No.	Identification result	Molecular formula	Ionization mode	Nucleation ratio	Retention time
1	1-Methylhistamine	C_6_H_11_N_3_	M + H	126.1025	0.98
2	L-Carnitine	C_7_H_15_NO_3_	M + H	162.1124	1.12
3	1-Methyladenosine	C_11_H_15_N_5_O_4_	M + H	282.1195	1.46
4	Phenylalanine	C_9_H_11_NO_2_	M + H	166.1003	1.61
5	Riboflavin	C_17_H_20_N_4_O_6_	M + H	377.1448	2.29
6	Cortisol	C_21_H_30_O_5_	M + H	363.2168	2.83
7	3-Indoleacrylic acid	C_11_H_9_NO_2_	M + H	188.0706	3.62
8	Glucose	C_6_H_12_O_6_	M − H	179.0560	0.66
9	Isocitrate	C_6_H_8_O_7_	M − H	191.0210	0.68
10	2-Oxoglutarate	C_5_H_6_O_5_	M − H	145.0143	0.68
11	Citrate	C_6_H_8_O_7_	M − H	191.0206	1.25
12	Atrolactic acid	C_9_H_10_O_3_	M − H	165.0563	2.43

## Data Availability

The data sets analyzed during the current study are available from the corresponding author on reasonable request.
